# Beyond the lungs: patients’ experiences of musculoskeletal symptoms and manual therapy in cystic fibrosis care – A qualitative interview study

**DOI:** 10.1080/10669817.2025.2505096

**Published:** 2025-05-14

**Authors:** Niklas Sinderholm Sposato, Jenny Danielsbacka, Marita Gilljam, Louise Lannefors, Kristofer Bjerså, Monika Fagevik Olsén

**Affiliations:** a Department of Health and Rehabilitation, Institute of Neuroscience and Physiology, Sahlgrenska Academy, University of Gothenburg, Gothenburg, Sweden; b Department of Physiotherapy, Sahlgrenska University Hospital, Gothenburg, Sweden; c Department of Respiratory Medicine, Sahlgrenska University Hospital, Gothenburg, Sweden; d Department of Surgery, Institute of Clinical Sciences, Sahlgrenska Academy, University of Gothenburg, Gothenburg, Sweden; e Family Medicine, School of Public Health and Community Medicine, Institute of Medicine, Sahlgrenska Academy, University of Gothenburg, Gothenburg, Sweden; f Primary Care, Närhälsan Majorna, Region Västra Götaland, Gothenburg, Sweden

**Keywords:** Musculoskeletal, pain, stiffness, manual therapies, cystic fibrosis

## Abstract

**Background:**

Cystic fibrosis (CF) is a severe hereditary disease that affects multiple organ systems. Among these, the musculoskeletal system is an under-explored area. This interview study aimed to explore experiences of musculoskeletal symptoms and of manual therapies as complementary care in this context.

**Methods:**

Semi-structured interviews were used to collect data from ten respondents. The data were subsequently analyzed through content analysis with an inductive approach in accordance with the method of Elo and Kyngäs.

**Results:**

The analysis resulted in three main categories; 1) Living with CF involves musculoskeletal health challenges, 2) Manual therapies impact daily life for people with CF, and 3) People with CF aspire for broader and more collaborative respiratory care.

**Conclusion:**

The respondents described musculoskeletal symptoms in and around the thoracic cage. They experienced symptom relief and increased body awareness following manual therapy interventions (MTI) and recommended that these methods be offered as complementary care to enhance quality of life.

## 1. Background

Cystic fibrosis (CF) is a rare and serious hereditary disease that affects multiple organ systems. The highest incidence occurs in individuals of European descent, with a prevalence of 1 in 3000 to 1 in 6000 live births. However, it also occurs among people from other ethnic backgrounds [1]. The respiratory system and its functions involve a complex interaction between visceral organs and the neuromusculoskeletal system. For people with CF, for whom the airways and lungs become severely compromised, treatment is directed toward the respiratory system from early childhood and continuously throughout life [2]. However, the research that has preceded and accompanied this essential treatment has mainly been focused on understanding and addressing the visceral aspects of respiration, i.e. the airways and the lungs, and to a lesser extent its musculoskeletal counterpart [3]. Due to the major medical advancements made over the past decades, life expectancy has now reached almost 50 years (and beyond for certain populations) [1,4]. However, the disease is still both life-shortening and highly life-limiting. The demographic shift that is occurring in CF presents new challenges, among which is a probable increase in various age-related conditions, including those affecting the musculoskeletal system [5,6].

Given the multifaceted nature of CF, care strategies in this context routinely include a combination of pharmacological treatments, dietary advice, psycho-emotional support, surgery, breathing exercises, airway clearance and individualized support for physical activity and exercise [7–10]. Conversely, manual therapy interventions (MTI), which are frequently used to address musculoskeletal complaints in other fields of practice, are rarely part of the care. MTI, combined with exercise or as a stand-alone intervention, has been demonstrated to have positive treatment effects in several musculoskeletal conditions, e.g. nonspecific neck pain [11,12] and chronic low back pain [13]. In terms of respiratory muscle involvement, the cervical and lumbar regions would be considered accessory, i.e. bodily areas that engage as the respiratory workload increases. For healthy individuals, the engagement of accessory respiratory muscles occurs during exercise. However, in CF and other lung diseases, the demand on these muscles is heightened in everyday life.

Considering the increase in life expectancy for people with CF and the challenges that this advancement has brought with it, it is crucial to expand on research priorities, and to a greater extent also include the patients’ lived experiences and perspectives [4]. By doing so, more individualized and effective clinical guidelines can be developed. Research on this group of patients’ experiences of musculoskeletal symptoms remains sparse. This also applies to experiences with MTI as a complementary treatment within this group. Although previous studies on manual therapies in CF care are both rare and often small-scale, they have indicated some positive effects [14]. These positive effects include improvements in pain perception and ease of breathing [14]. In summary, the limited amount of research in these areas makes it difficult to comment in a relevant manner on the musculoskeletal health needs of these patients. Hence, the aim of this study was to explore the lived experiences of musculoskeletal symptoms and the use of specific manual therapies for such symptoms among people living with CF.

## 2. Methods and materials

### 2.1. Participants

The study included adult patients (>18 years old) with CF enrolled at Sahlgrenska University Hospital’s CF Centre, Gothenburg, Sweden, who had received MTI between the years 2019 and 2022 as part of a pilot study [15]. The MTI primarily targeted the thoracic cage and musculoskeletal structures relevant to breathing. Secondary anatomical areas were included when clinically indicated. Recruitment for the current study commenced in January of 2022.

The inclusion criteria were adult patients, age >18 years who had presented with musculoskeletal stiffness and/or pain and who had received MTI in the form of passive joint mobilization and soft tissue treatment for eight consecutive weeks (one 30-minute MTI session per week). An invitation letter was sent out to all (*n* = 15) eligible patients who met the inclusion criteria. The letter contained study information and a request for written consent to participate. Patients who gave consent to participate (*n* = 10) were contacted by telephone to schedule the interview. A total of ten participants were ultimately included. Their demographic data are presented in Table 1.

**Table 1.** Demographic data of the respondents.

Characteristics	Number or mean (range)
Sex, female/male	4/6 respondents
Age	34.2 (20–51) years
Time from intervention to interview	8.4 (<1–29) months
Time spent in interview	28 (13–52) minutes

Author’s comment: A distinction has been made in this paper where individuals included in the study are initially referred to as ‘participants’ in the sections describing the recruitment process. As these individuals contributed their experiences in interviews, they are instead referred to as ‘respondents’.

### 2.2. Data collection

An initial interview guide was constructed by the first author, which was then revised following expert review by two researchers (MFO and JD) who had experience of the chosen methodology. The final interview guide was structured around five open questions with a series of secondary, more confined follow-up questions. The questions were formulated with the ambition to provide the respondents with freedom to broadly reflect on their experiences concerning the overall aim of the study.

A total of ten semi-structured interviews were carried out between the 10^th^ of February and the 12^th^ of December 2022. The interviews began with the request *‘Describe how you experience your symptoms in muscles and joints in and around your chest’*, after which follow-up questions were asked. The subsequent themes covered by the interview guide were subsequently explored in a corresponding manner. The interviews were conducted via video link at a time chosen by the respondent. All interviews were audio-taped, coded, and transcribed verbatim. After the final interview had been completed, the analysis process was initiated.

### 2.3. Data analysis

**Figure 1.**
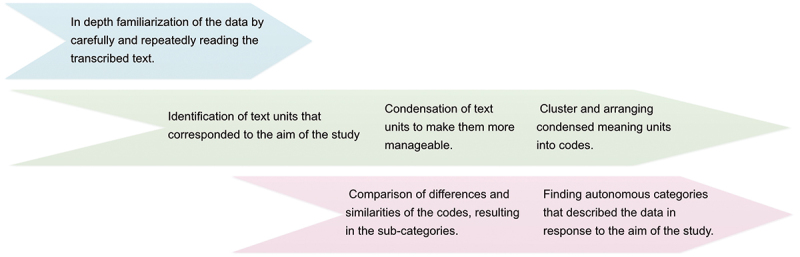
The inductive analysis process, guided by and adapted from Elo and Kyngäs [16].

A qualitative content analysis using an inductive approach was carried out using the steps outlined by Elo and Kyngäs [16]. This step-by-step process, which proceeds from a preparation phase through an organization phase and finally to a result phase, is presented in Figure 1. Initially, the data were analyzed by two of the authors (NSS and JD). These initial analyses were then reviewed and added to by a third author (MFO). Supported by this second analysis, the material was revised a final time, leading to identification of the sub-categories and categories that aligned with the study’s aim. An example of the inductive analysis process is presented in Table 2.

**Table 2.** Example of the inductive analysis process.

Meaning unit	Condensed meaning unit	Code	Sub-category	Category
*‘Sometimes it feels like I have a pain in my heart and sometimes like I have a pain somewhere in my lungs’*	*‘It feels like I have pain in my heart and sometimes somewhere in my lungs’*	Experience of pain in heart and lung	Pain and stiffness are part of everyday life	Living with CF involves musculoskeletal health challenges
*‘Breathing becomes kind of a challenge for the body sometimes, the whole respiratory gymnastics, the coughing’*	*‘Breathing becomes a challenge for the body’*	Breathing as a physical challenge	Breathing and breathing exercises are physically demanding	
*‘For me, the thorax is primarily a prerequisite for the lung much more than it is a separate issue’*	*‘The thorax, a prerequisite for the lung and not a separate area of concern’*	Viscero-Somatic interdependence	Proper thoracic function is essential for respiration	

## 3. Results

**Figure 2.**
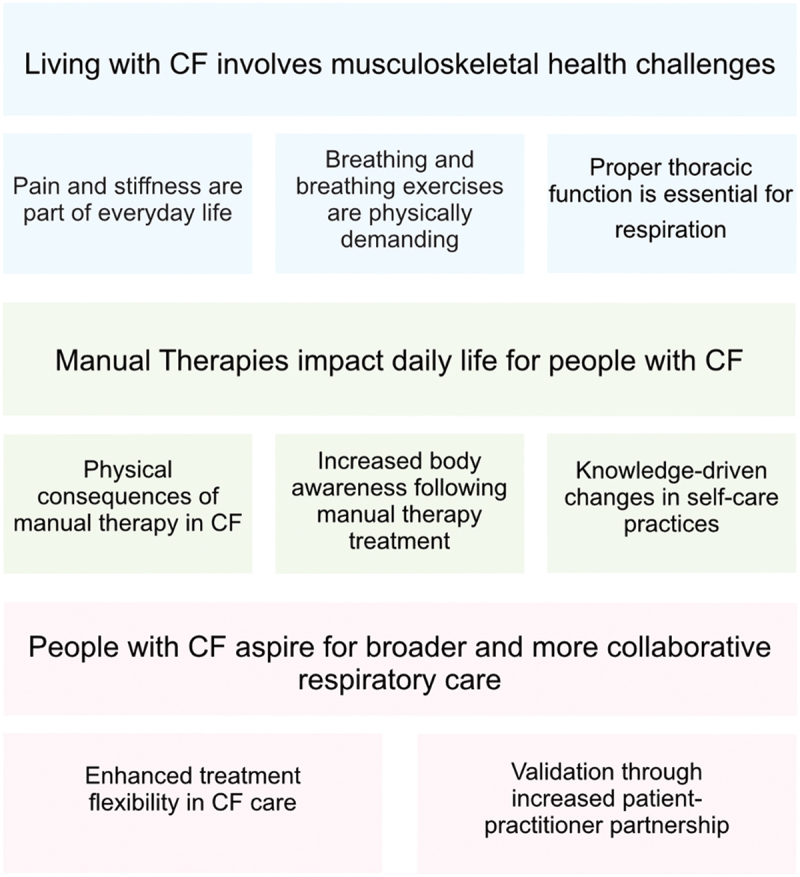
Main categories and sub-categories yielded by the content analysis.

The analysis resulted in three main categories and eight sub-categories, which are presented in Figure 2. The quotes presented are intended to highlight the results.

The presentation below follows the structure illustrated in Figure 2, with each main category introduced alongside its respective sub-categories.

### 3.1. Living with CF involves musculoskeletal health challenges

The respondents described how living with CF entails ongoing physical challenges. These challenges include musculoskeletal pain and stiffness, which both affect and are affected by breathing. The respondents also stated that although they recognized the importance of the musculoskeletal aspects of breathing, they had not previously given them much thought. Regarding the interplay between the visceral and musculoskeletal components, they described an inside-out perspective in their way of perceiving breathing. They regarded the ‘outside’ as an important prerequisite for the functioning of the ‘inside’, even if it was not necessarily their main focus of attention.

#### 3.1.1. Pain and stiffness are part of everyday life

The interviews revealed that symptoms of pain and decreased mobility were common among the respondents. Furthermore, these symptoms were not limited to the thorax, but rather to several bodily areas. Of these secondary musculoskeletal areas of respiration, the shoulder girdle, cervical area, and head, including headaches, were more often referred to by the respondents. In periods of pulmonary exacerbation, the musculoskeletal symptoms were reported to increase.

The whole upper body is tense and aches a lot … as part of the whole I also have a lot of headaches.

When I get sick, I get blockages and I become crooked, and yeah, I experience it as if my thorax gets stuck.

#### 3.1.2. Breathing and breathing exercises are physically demanding

Respondents described that breathing as constrained by the underlying disease was demanding, both in terms of continuous coughing, for many, and in terms of airway clearance and breathing exercises. These physical demands, which could increase in periods of exacerbation, were not confined to the thorax but instead affected the body as a whole.

I cough quite a bit and I work a lot with the muscles during respiratory exercises, so I think there are more parts of the body that are affected as well.

When you have been sick for a long period, it kind of feels like the chest gets bigger or something like that, and I kind of think it does, because the air doesn’t come out properly.

#### 3.1.3. Proper thoracic function is essential for respiration

It was emphasized by the respondents that the structural and functional integrity of the thorax was important. This consideration was primarily expressed in terms of the thorax acting as an enabler or disabler for the lungs and proper breathing. Among these respondents, however, one could notice a certain shift in perspective after undergoing a period of treatment with manual therapy, where the focus was on the musculoskeletal system. Several of the respondents reported new behaviors, something that will be covered in more detail in the upcoming results sections.

In a way, the thorax is just there to hang the lungs in, and then they have to be as good as possible to counteract the decay of the lung, so to speak.

Yes, I mean, if they (the lungs) don’t have space to move, then they can’t breathe properly.

### 3.2. Manual therapies impact daily life for people with CF

Overall, the respondents’ experiences of manual therapy were positive. It appeared that these treatments contributed to a decrease in physical symptoms, an increase in body awareness and beneficial changes in self-care practices. These results indicate that manual therapy can play a positive role, not only physically but also by strengthening the patient’s knowledge and thereby their independence.

#### 3.2.1. Physical consequences of manual therapy in CF

The respondents described, on one hand, the impact on symptoms such as reduced pain, increased mobility, and ease of breathing during the treatment period, and on the other hand, effects such as being able to mobilize mucus more easily. Furthermore, an overall feeling of being more satisfied with their body was expressed.

I have been satisfied with my body these past few months, like, it hasn’t bothered me as it usually does.

I was in less pain, it felt easier to breathe afterwards, and I was able to clear more mucus.

#### 3.2.2. Increased body awareness following manual therapy treatment

The interviews revealed that the treatment period with manual therapy, consisting of non-thrust passive joint mobilization and soft tissue techniques directed at both primary and secondary musculoskeletal areas related to respiratory function, increased body awareness in the current group of respondents. The musculoskeletal aspects of respiration were for many something that had previously been given a more secondary focus. Through the manual therapy sessions, which primarily aimed to affect musculoskeletal health, the respondents were able, to a greater extent, to also consider these aspects of their health.

I have worked my whole life from the inside out, but now I’m doing it a bit more, maybe like, from the outside in.

More aware in general, like, of what one does and doesn’t do and how one takes care of oneself.

#### 3.2.3. Knowledge-driven changes in self-care practices

The manual therapy sessions led to an increased focus on the musculoskeletal aspects of the respiratory system. This shift in focus resulted in respondents spending more time on mobilizing exercises and intensifying their strength training. This increased insight into the role of the muscles and joints not only made each respondent more aware of their body but also strengthened their self-confidence in relation to it.

A lot of movements every morning to, like, warm up the body, and then I put a bit of extra focus specifically around the thoracic area to try to keep it as mobile as possible.

### 3.3. People with CF aspire for broader and more collaborative respiratory care

The interviews revealed a desire for even more inclusive and flexible care. This could include access to more treatment options but also more individualized measures and a strong patient-practitioner collaboration.

#### 3.3.1. Enhanced treatment flexibility in CF care

Regarding implementation, the respondents regarded manual therapy as a beneficial addition to their musculoskeletal health regimen, rather than a stand-alone treatment. They suggested that manual therapy could be offered periodically following periods of exacerbation, as a continuous component to facilitate training, or even as a preventive measure. Regardless of the potential application, the respondents emphasized that this intervention should be available for those interested, as part of a personalized care approach.

For me, it has worked perfectly, I’ve tried so many other treatments, so why not include this as well? After all, I’m open to anything that has a positive effect.

A good complement to other things we do… especially since we do a lot of breathing exercises.

#### 3.3.2. Validation through increased patient-practitioner partnership

The respondents experienced it as positive when caregivers asked open questions about their well-being, when they offered physical assistance with the treatment, and when knowledge was shared that increased their own understanding. Furthermore, the feeling of doing things together was described as relieving. This result was not unique to the treatment period within the study, but also something that the respondents had experienced in previous care situations.

He asked a lot “How does it feel today”? “Is there anything special you want to bring up” or something like “Have you thought about anything recently”, and it felt very good to be asked such questions because it might not be something you think about specifically but something that emerges over time.

A similar feeling to when I go to the physiotherapist at the CF clinic, and he or she, depending on who is working there, just like this, places a hand on, and says “Now we’re going to exhale”, like, assists and exhales, it’s so helpful, we’re sharing the burden.

## 4. Discussion

In the present study, patients’ experiences of musculoskeletal health aspects were explored. The results showed that the respondents lived with pain and stiffness in muscles and joints as part of their daily life. These symptoms were most prevalent in the thorax, neck and head but could affect the body as a whole. As already stressed, musculoskeletal health is generally underrepresented in CF research, although some studies have begun to address associated challenges [8,14,15]. Within this already underrepresented field of musculoskeletal health, the thorax is in turn a generally overlooked area [17]. Previous studies have shown that thoracic pain often does not occur in isolation, but among those who seek care for cervical or low back pain, approximately 41% of men and 36% of women also reported pain from this bodily area [18]. These previous results align with the experiences reported in the current study. As such, this knowledge may enhance our understanding of how pain experiences across different bodily regions may be interrelated. However, one should be careful not to engage in ‘anatomical possibilism’ [19] by speculating, or by asserting specific clinical implications of such bodily connections without further investigation.

In treatment guidelines for musculoskeletal conditions, non-pharmacological interventions such as MTI together with physical exercise, are recommended [20]. This aligns with the findings of the current study, where the respondents experienced decreased pain and increased mobility following such complementary treatments. In addition, the respondents said that during the treatment period, they gained increased body awareness and adjusted their self-care routines in ways that benefited them. The therapeutic encounter for health professionals who provide MTI often also includes counseling related to lifestyle, exercise, physical activity, pain management and plans for multidisciplinary care strategies [21]. It is therefore difficult, and perhaps inappropriate to try to completely make the separation between the therapy and the therapeutic encounter when discussing positive consequences of treatment. Notably, as MTI did not replace other care during the previous study period, situations arose where respondents experienced improved outcomes from other interventions as well, for example, the ability to engage in physical activities and exercise with greater ease. This experience, which needs to be further researched, could be an indication that MTI can serve as an initial support or complement to other physiotherapeutic interventions, or can even be an integral part of the overall care plan.

Adopting a more nuanced and integrated approach to MTI certainly entails difficulties in solely assessing the physical act that the treatment entails. However, by acknowledging the multifaceted nature of the patient-practitioner interaction, a more realistic and clinically relevant understanding can be obtained. Furthermore, such an approach is consistent with an ambition to establish a strong therapeutic alliance, something that the respondents valued. This is also in line with previous research that has indicated that reduced musculoskeletal pain and treatment satisfaction correlate with a good patient-practitioner relationship [22].

In terms of strengths and limitations, a main strength is the topic of investigation itself. Musculoskeletal health and the effects of MTI are both relatively under-explored areas within CF care. The opportunity to gain knowledge on these areas through patient experiences allows for the refinement of future research, which may ultimately lead to new health care strategies and the discovery of previously unrecognized impacts of MTI, such as increased body awareness. However, as the results from this study are based on the experiences of only one group of people with CF from just one hospital, it is not possible at this time to draw any decisive conclusions from which clinical guidelines can evolve. Furthermore, all treatments that preceded the interviews were provided by a single practitioner. This introduces a potential challenge in separating the effects of the intervention from those attributable to the therapeutic encounter in general. As previously mentioned, this is a factor that should be acknowledged but also accepted as inherent to most forms of therapy involving person-to-person interaction. However, to reduce the risk of bias, all interviews were conducted by a person who was not known to the respondents. The interval between the intervention and the interviews differed among participants, by an average of 8.4 months. This was partly due to delays caused by the COVID-19 pandemic, which impacted research activities involving vulnerable populations, including people with CF. This variation may have affected recall, but it may also have allowed respondents to contemplate the potential long-term effects of the intervention.

Trustworthiness, which is a central concept in qualitative research [16,23], was strengthened in this study by the three researchers reaching consensus on the analysis process and the results. By involving several people in the interpretation of the data, greater certainty could be reached regarding whether the analyses represented the participants’ experiences in a reliable way. Furthermore, the research process was carefully and transparently documented, which facilitates an assessment of both the credibility and transferability of the findings. Something that could increase credibility further but was not applied in this study would be to give the respondents the opportunity to review the interpretations that were made of the collected data [24]. However, such a methodological choice comes with its own challenges such as time and resource limitations, participants’ availability, and a risk of influencing their original opinions.

Finally, in recent years, the implementation of new and highly efficient modulator treatments has begun. These pharmacological therapies target the underlying CFTR protein defect in CF. At the time of the intervention study that preceded this interview study, these treatments had not yet been implemented in Sweden. However, for many, the effects demonstrated so far for these modulator treatments have been positive [4,25]. This development is being watched closely, as significant change in musculoskeletal and other areas of CF care is likely.

## 5. Conclusion

The respondents described living with CF as being affected daily by stiffness and pain in the muscles and joints of the thoracic cage and associated body areas supportive of respiration. They experienced that MTI reduced these symptoms and wished that such methods could be offered as an optional adjunct to other care to better support individualized management. As in many other countries, Swedish CF care has long been characterized by multidisciplinary collaborations and individualized care. Considering the demographic changes among people with CF, it may be necessary to further adapt this approach to effectively address the specific needs of this group regarding their musculoskeletal health. For MTI to be part of such adaptation, future research must first explore the potential benefits of these treatments through full-scale studies, including, but not limited to randomized clinical trials.
